# Multidisciplinary Decision-Making and Integrated Strategies in Stage III Non-Small Cell Lung Cancer: Exploring Clinical Reasoning in Therapeutic Choices

**DOI:** 10.3390/jcm15103752

**Published:** 2026-05-13

**Authors:** Paolo Borghetti, Fabiana Vitiello, Niccolò Giaj-Levra, Alessio Bruni, Fabiana Cecere, Marco Chiappetta, Patrizia Ciammella, Francesco Guerrera, Antonio Mazzella, Michele Montrone, Alessandro Russo, Vieri Scotti, Diego Signorelli, Stefano Vagge, Filippo Lococo

**Affiliations:** 1 Radiation Oncology Department, ASST Spedali Civili and University of Brescia, 25123 Brescia, Italy; paolobor82@yahoo.it; 2 Department of Pneumology and Oncology, Monaldi Hospital A.O. Dei Colli, 80131 Naples, Italy; fabianavitiello@libero.it; 3 Advanced Radiation Oncology Department, IRCCS Sacro Cuore Don-Calabria, 37024 Negrar di Valpolicella, Italy; 4 Department of Medical and Surgical Sciences for Children & Adults, University of Modena and Reggio Emilia, 41124 Modena, Italy; bruni.alessio@aou.mo.it; 5 Radiotherapy Unit, University Hospital of Modena, 41124 Modena, Italy; 6 Medical Oncology 2, IRCCS Regina Elena National Cancer Institute, 00144 Rome, Italy; fabianaletizia.cecere@ifo.it; 7 UOC di Chirurgia Toracica Fondazione Policlinico Universitario A. Gemelli IRCCS, 00168 Roma, Italy; marcokiaps@hotmail.it (M.C.);; 8 Radiation Oncology Unit, Azienda USL-IRCCS di Reggio Emilia, 42123 Reggio Emilia, Italy; patrizia.ciammella@ausl.re.it; 9 Department of Cardio-Thoracic and Vascular Surgery, Azienda Ospedaliera-Universitaria Città Della Salute e Della Scienza di Torino, 10126 Torino, Italy; francesco.guerrera@unito.it; 10 Division of Thoracic Surgery, IEO European Institute of Oncology, IRCCS, 20141 Milan, Italy; 11 Medical Thoracic Oncology Unit, Istituto Tumori Giovanni Paolo II, 70124 Bari, Italy; 12 Medical Oncology Department, Humanitas Istituto Clinico Catanese, 95045 Misterbianco, Italy; 13 Radiation Therapy Unit, Department of Oncology, Careggi University Hospital, 50134 Firenze, Italy; 14 Niguarda Cancer Center, ASST Grande Ospedale Metropolitano Niguarda, 20162 Milan, Italy; diego.signorelli@ospedaleniguarda.it; 15 Radiotherapy Department, E.O. Ospedali Galliera, 16128 Genova, Italy

**Keywords:** NSCLC, survey, multidisciplinary, local treatment, locally advanced

## Abstract

**Background/Objectives**: Stage III non-small cell lung cancer (NSCLC) is a heterogeneous and clinically challenging disease. Despite therapeutic advances, decisions regarding resectability and treatment sequencing remain complex. Multidisciplinary discussion (MDD) is increasingly recognized as key to personalized, evidence-based care. **Methods**: The “Integrate 6.0” conference gathered approximately 90 lung cancer specialists, including oncologists, thoracic surgeons, and radiation oncologists, divided into mixed groups simulating multidisciplinary team (MDT) meetings. Groups reviewed complex clinical cases, supported by facilitators providing concise, evidence-based updates. A pre-event survey explored MDT structure and dynamics across institutions. **Results**: The survey highlighted considerable variability in MDT composition and practices. Most participants had significant involvement in thoracic oncology. Discussions revealed higher consensus in straightforward cases, while complex stage III scenarios—especially with driver mutations or bulky nodal disease—required more nuanced, collaborative decision making. Key topics included neoadjuvant chemoimmunotherapy, surgery in borderline resectable cases, and managing immune-related toxicities. **Conclusions**: “Integrate 6.0” effectively connected theoretical knowledge with real-world practice through interactive, multidisciplinary dialogue. It underscored the vital role of MDD in managing complex stage III NSCLC and the need for adaptable treatment strategies. Future conferences should assess MDD’s impact on outcomes and expand participation to include molecular pathologists and geriatricians.

## 1. Introduction

Lung cancer is the leading cause of cancer-related mortality, accounting for more deaths than colorectal, breast, and prostate cancers combined [1]. Non-small cell lung cancer (NSCLC) represents the most common histological subtype. In recent years, the introduction of immunotherapy and targeted therapies has significantly improved outcomes in both unresectable locally advanced and resectable disease [2,3,4,5,6,7,8]. Stage III NSCLC is a highly heterogeneous disease, characterized by a substantial risk of recurrence, with more than 60% of patients experiencing either distant metastases or local relapse following surgery. A 5-year overall survival rate ranging from 13% to 36% was reported [9]. Accurate staging is therefore critical and it is recommended by international guidelines to include brain imaging, PET-CT and a histopathological confirmation of mediastinal nodal involvement when indicated (e.g., PET-positive mediastinal lymph nodes, tumors larger than 3 cm or hilar nodal involvement (N1)), using EndoBronchial Ultrasound (EBUS) or Endoscopic Ultrasound (EUS) [10]. In stage III NSCLC a major challenge is the assessment of resectability. Early evaluation by a multidisciplinary team (MDT), including all specialists involved in lung cancer management, is strongly recommended to define the most appropriate treatment strategy [11,12]. Although resectability is typically determined by thoracic surgeons, it remains a gray zone, with decisions often influenced by individual expertise and institutional experience. To address this variability, the EORTC Lung Cancer Group conducted a survey involving members of international lung cancer organizations to establish consensus criteria for clinical trial design in stage III disease. A multidisciplinary panel comprising thoracic surgeons, medical oncologists, radiation oncologists, pulmonologists and other specialists, primarily from high-volume centers, assessed the resectability of 37 TNM subsets. Consensus was achieved for several scenarios; for example, N3 disease, invasive N2 (with infiltration of surrounding structures) and bulky N2 (nodal short axis > 3 cm) were generally considered unresectable. However, agreement was lacking for more controversial presentation, such as single-station T4N2 disease and selected cases of multistation N2 involvement [13,14]. For patients with resectable stage III NSCLC, surgery remains a cornerstone of treatment being typically combined with systemic therapy. Both neoadjuvant or adjuvant platinum-based chemotherapy showed comparable benefits, with an absolute improvement in the 5-year overall survival rate by approximately 5% [15,16]. More recently, immunotherapy and targeted therapies have shown efficacy in the adjuvant setting [6,7,17,18] whereas postoperative radiotherapy has not demonstrated a clear benefit in disease-free survival [19]. Immunotherapy has also been investigated in the neoadjuvant and perioperative setting. Phase III trials evaluating neoadjuvant chemo-immunotherapy or perioperative approaches (neoadjuvant chemotherapy followed by adjuvant immunotherapy) have met key endpoints, including event-free survival and pathological complete response rates. The KEYNOTE-671 trial also demonstrated an overall survival (OS) benefit [20,21,22,23]. Ongoing phase III trials are exploring the role of targeted therapies in the neoadjuvant setting. However, similar to advanced disease, patients with certain oncogenic driver alterations unrelated to tobacco exposure, such as EGFR mutations or ALK rearrangements, derive limited benefit from the addition of immunotherapy in the perioperative or adjuvant treatment [16,22]. Therefore, comprehensive molecular profiling, particularly in non-squamous histologies, is essential prior to treatment initiation. In unresectable stage III NSCLC, the standard of care remains concurrent chemoradiotherapy, or sequential treatment when concurrent is not feasible [24]. Previous attempts to improve outcomes with chemotherapy or targeted therapy in unselected populations did not demonstrate an OS benefit [25,26].

The PACIFIC trial revolutionized clinical practice, demonstrating significant improvements in both progression-free survival (PFS) and OS with durvalumab consolidation therapy administered for up to one year following concurrent chemo-radiotherapy [4]. These findings have been confirmed in real-world studies, including PACIFIC-R [27,28]. However, subgroup analyses suggest that patients with EGFR-mutated NSCLC do not obtain the same benefit from consolidation immunotherapy [29]. In this population, osimertinib has shown a statistically and clinically significant improvement in PFS compared with placebo after concurrent or sequential chemo-radiotherapy and is administered until disease progression or unacceptable toxicity [5].

Given the complexity and rapid evolution of the therapeutic landscape, stage III NSCLC remains a topic of active discussion at local, national, and international levels. Several unmet clinical needs remain open and the multidisciplinary discussion is essential to integrate diverse expertise and optimize patient management. In this context, the 6th edition of the “Integrate” project hosted a two-day meeting in October 2024 entitled “Integrated strategies in the management of lung cancer”. The event brought together medical, radiation oncologists, and thoracic surgeons to exchange perspectives and discuss current treatment approaches for stage III NSCLC. Recognizing the need for more interactive and practice-oriented education formats, the meeting adopted a model based on structured, small-group discussion and case-based simulation. This approach aimed not only to update knowledge but also to enhance multidisciplinary decision-making skills and foster cross-specialty collaboration. This article aims to summarize the key outcomes of the meeting, which was conducted using a structured and comparative discussion format to facilitate comparative discussion and enhance collective expertise on this debated topic.

## 2. Materials and Methods

The primary aim of the meeting was to promote the critical role of the multidisciplinary approach in the management of locally advanced stage III NSCLC patients and to provide practical training in collaborative decision making. The conference was designed for healthcare professionals actively involved in lung cancer treatment and routinely participating in multidisciplinary tumor boards including thoracic surgeons, medical and radiation oncologists. Clinicians were invited by the Scientific Board to participate in the meeting, and all invitees attended and were included in the subsequent activities. Participants were selected based on their active involvement in thoracic oncology and regular participation in multidisciplinary discussions. To ensure a representative sample, predefined selection criteria were applied. In particular, clinicians were required to dedicate the majority of their clinical activity (at least 70%) to lung cancer management. Efforts were made to achieve a broad geographic representation across the national territory (Northern, Central, and Southern regions) and to include both senior and younger clinicians with extensive professional experience, in order to foster a comprehensive and multidimensional discussion. The higher proportion of radiation oncologists among participants reflects the organizational framework of the meeting, which was coordinated within a radiation oncology network. Nevertheless, particular attention was paid to ensuring adequate and representative participation of medical oncologists and thoracic surgeons. Moreover, each discussion group was structured to include clinicians from different specialties, thereby preserving the multidisciplinary nature of the case discussions. No formal exclusion criteria were pre-defined.

One month prior to the meetings, all invited participants were requested by the Scientific Board to complete an anonymous survey consisting of 36 questions focused on the multidisciplinary management of lung cancer patients. Participation was strongly recommended, and all responses were collected anonymously. The questionnaire was administered in Italian to ensure clarity and comprehension. The survey was initially developed by five radiation oncologists from the Scientific Board and subsequently reviewed and refined by the remaining board members, including medical oncologists and thoracic surgeons, to ensure content validity, clarity, and multidisciplinary relevance. The survey was distributed via e-mail together with a cover letter providing background information on the questionnaire. The questionnaire comprises two sections: (1) demographic and professional characteristics of the respondents; (2) organization and functioning of the multidisciplinary team. The questions were designed to be multiple-choice, concise, easy to interpret, and aligned with the aim of the meeting, while minimizing respondent burden. Several items were based on clinical scenarios to explore decision-making processes in routine practice. Only fully completed questionnaires were included in the analysis, as all survey items were mandatory and participants were required to complete each question before proceeding to the next.

At the meeting, participants were divided into 8 groups of about 10 clinicians each (in total, 85). Every group (from A to H) was structured to ensure a balanced representation of different specialties. Groups were assigned to separate tables and rooms to allow independent discussion without external influence. The activities were coordinated by two faculty members per group, who acted as facilitators.

On the first day, four clinical cases were presented to each group by a designated speaker, who outlined three key decision-making points and guided the ensuing discussion (Supplementary File Supplement S1). During the case discussion, a facilitator from the faculty managed the interaction between clinicians, while a second one systematically recorded predefined variables using a structured electronic data collection form developed by the Scientific Board.

These variables concerned the entire discussion, and particularly the group’s approach to the decision-making points. The data collected were:

(A) The speciality of the group leader.
(B) The rationale behind the choices:
  - Evidence-based medicine.
  - Previous experiences.
  - Available resources and adequate skills.
(C) Duration of the discussion from the presentation of the clinical case to the group’s decision:
  - 1 to 5 min.
  - 6 to 10 min.
  - 11 to 15 min.
  - 16 to 20 min.
(D) Possible obstacles to the final decisions due to:
  - Evidence-based medicine.
  - Previous experiences.
  - Available resources and adequate skills.
(E) The simulation of a multidisciplinary team (MDT) was analyzed, noting:
  - Whether a single physician led the discussion.
  - Whether 2 to 3 physicians led the discussion.

Whether all of the participants contributed to the discussion, immediately after the clinical case discussion, the latest therapeutic updates (related to the presented clinical case) were illustrated by a speaker through a ten-minute frontal presentation.

On day 2 of the meeting, the collected information was reported in plenary room through a graphic representation elaborated by a communication agency.

Survey data were considered in a descriptive analysis using SYSTAT statistical software 13.2 (Systat Software, Point Richmond, CA, USA). Consensus was defined as a priori agreement among at least 70% of participants within each discussion group. All clinical cases and multiple choices are summarized in Supplementary Figure S1.

## 3. Results

### 3.1. Survey

A total of 85 participants attended the Integrate 6.0 meeting, including 30 radiation oncologists, 30 medical oncologists and 25 thoracic surgeons. The pre-meeting survey, distributed before the event to a broader group of clinicians, was completed by 94 respondents, comprising 33 radiation oncologists, 25 medical oncologists, 27 thoracic surgeons, 5 pneumologists, 1 pathologist, 1 general surgeon, 1 vascular surgeon and 1 medical physicist (Supplementary Figure S2).

Regarding the survey respondents, most worked in academic or non-academic public Institutions, primarily located mostly in Northern or Central regions of Italy (Supplementary Figure S3). Participants’ professional experience (years of practice) and the proportion of time dedicated to lung cancer management are reported in Supplementary Figure S3. Multidisciplinary teams included a core group composed of thoracic surgeons (92%), medical (94%) and radiation oncologists (94%), pneumologists (89%), radiologists (86%) and pathologists (76%). Other professional figures were less frequently represented, including nuclear medicine physician (60%), interventional radiologist (53%), molecular biologist (31%) and nurse (23%). In 70% of cases, all MDT members belonged to the same institution.

Diagnostic work-out procedures were generally managed by a pneumologist, thoracic surgeon and medical oncologist in 43%, 31% and 26% of cases, respectively. Interestingly, multidisciplinary groups tended to discuss cases across all disease stages, rather than limiting discussion to locally advanced or more complex cases. The survey responders reported that their multidisciplinary teams discussed fewer than 10, 10 to 20, more than 20 cases weekly in 19%, 61% and 20%, respectively.

### 3.2. Clinical Case Discussion

**Figure 1:**
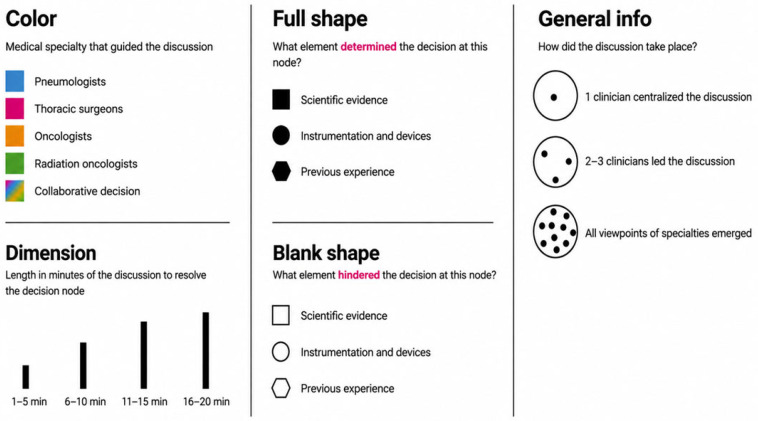
Legend used for the graphical representation of multidisciplinary discussions during the clinical case simulation.

**Figure 2:**
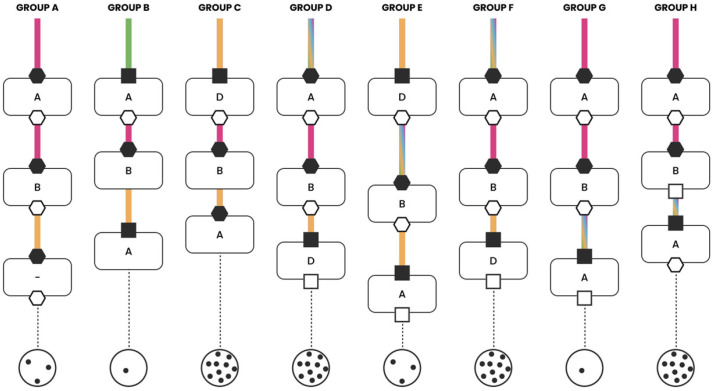
NSCLC CT4N0M0, Eligible to for Pneumonectomy.

**Figure 3:**
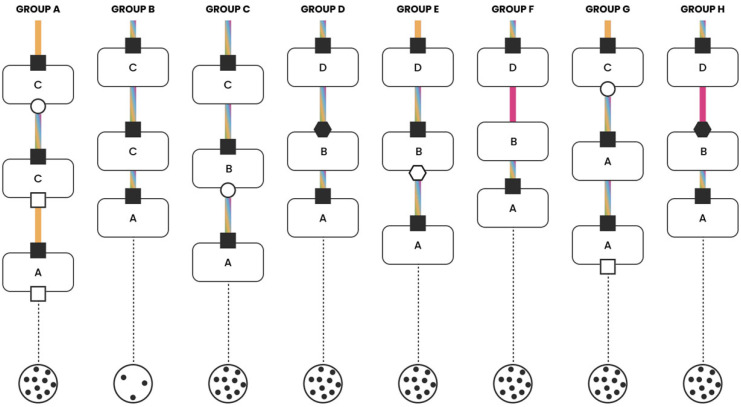
NSCLC cN2 multistation with PD-L1 > 50%.

**Figure 4:**
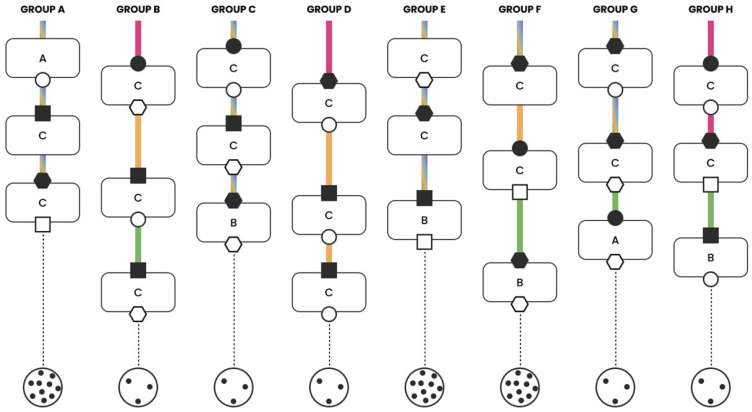
NSCLC stage III multiple N2 with driver mutation.

**Figure 5:**
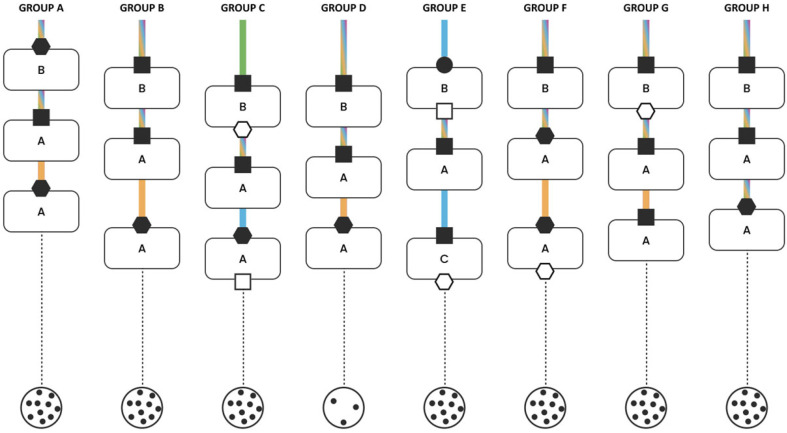
NSCLC stage III bulky N2 non-oncogene-addicted.

The clinical cases were discussed interactively within the multidisciplinary groups (Figure 1). Each group was coordinated by two facilitators: one moderated the discussion, while the second recorded predefined variables to document the decision-making process and generate graphical summaries (Figure 2, Figure 3, Figure 4 and Figure 5). The outcomes of the four emblematic clinical cases and the related graphics are reported in the following graph (shown according to the legend in the figure).

### 3.3. Case 1: NSCLC CT4N0M0, Eligible for Pneumonectomy

A patient diagnosed with left lung T4N0 adenocarcinoma was deemed eligible for left pneumonectomy. Three decision-making points were discussed, all related to the therapeutic approach.

As shown in Figure 2, the analysis of the discussion outcomes revealed a high level of consensus, especially for the second and third decision-making points. However, in the first decision point, different viewpoints emerged regarding the initial management strategy. Only two groups reached a shared decision, while in the remaining groups, the opinion of a single specialist prevailed. The discussion of this initial decision point lasted on average between 11 and 15 min in most groups. The decisions taken were supported either by scientific evidence (three out of eight groups) or by previous clinical experience (five out of eight groups). At the second decision point, there was a notable degree of consistency among the various groups, with the thoracic surgeon’s perspective generally prevailing. An exception was observed in one group, where the therapeutic decision was the result of a highly collaborative discussion, involving all participating professionals. In examining the rationale behind the decisions, prior experience with similar played a major/predominant role, rather than data from the literature or the availability of specific technologies. The duration of this discussion was shorter than the first, averaging between 6 and 10 min. During the third and final decision point, a substantial uniformity in therapeutic indications was observed across the groups, with the medical oncologist often guiding the final choice. Discussions at this stage were generally brief, with decisions being influenced by scientific evidence (six out of eight groups) and partly by prior clinical experience (two out of eight groups).

A global analysis of the discussions across all decision points revealed that in only four out of eight groups the perspectives of all involved specialists were represented. In the remaining four groups, the discussion was led and managed by a single professional or, at most, a subset of three specialists. A graphical summary is presented in Figure 2.

### 3.4. Case 2: NSCLC cN2 Multistation with PD-L1 >50%

A female patient was diagnosed with stage IIIB (cT3N2) lung adenocarcinoma, presenting with multistation mediastinal lymph node involvement and PD-L1 expression greater than 50%. Three decision-making points were proposed during the discussion: one about the diagnostic management (specifically, the necessity of biomolecular analysis) and two related to the therapeutic approach.

The first decision point was characterized by a high degree of homogeneity, with decisions being largely shared among participants; in only two groups the decision was strongly guided by the medical oncologist. The discussion was brief (lasting 1–5 min), and in all groups, the decision was grounded in scientific evidence.

The second decision point also prompted relatively brief discussion yet revealed greater heterogeneity in the treatment choices. Two groups opted for exclusive chemo-radiotherapy, five groups for a neoadjuvant treatment followed by surgery, and one group for upfront surgery followed by adjuvant therapy. Despite this heterogeneity, the decisions were generally shared within each group, drawing primarily based on data from the literature (five out of eight groups), with prior clinical experience playing a more marginal role (two out of eight groups). In only two groups a single professional, the thoracic surgeon, specifically predominated in guiding the discussion and making the final decision.

The final discussion point was again briefly (1–5 min), with a high degree of collaboration across groups, resulting in a largely uniform decision.

In summary, the management of this clinical case was characterized by a high level of collaboration across all groups, with the vast majority of decisions at each crucial point being made jointly. A graphical summary is presented in Figure 3.

### 3.5. Case 3: NSCLC Stage III Multiple N2 with Driver Mutation

A sixty-eight-year-old man has lung EGFR mutated (L858R) adenocarcinoma, staged as cT4N2 (IIIB), featuring multistation mediastinal lymph node involvement.

The first decision point was focused on the diagnostic workup. In all groups but one, the discussion was brief (ranging from 1 to 10 min). The decision-making process was collaborative and shared in the majority of groups (five out of eight).

Regarding the second decision point (therapeutic strategy), there was substantial agreement in therapeutic choices across groups, with the decision reached rapidly in most cases. However, shared and collaborative decision making occurred in only four out of eight groups; in the remaining groups, a single specialist led the discussion and guided the therapeutic decision. The rationale for the chosen treatment was predominantly based on scientific evidence (seven out of eight groups). The third decision point was similarly marked by brief discussions and a high degree of consistency in the therapeutic choices across the groups. Nonetheless, a collaborative decision-making process was evident in only two out of eight groups, while the discussion and final decision were influenced primarily by a single professional in the others. As in the previous decision points, choices were generally supported by scientific evidence and clinical experience. Only in one group was the availability of appropriate technology cited as a contributing factor.

In summary, analysis of the third clinical case indicates a notable degree of homogeneity in decision making, particularly at the second and third points. While multiple perspectives emerged during the discussions, in five out of eight groups the conversation and final choices were primarily led by a limited number of participants (1–3), suggesting that not all the specialists’ points of view were adequately represented. A graphical summary is presented in Figure 4.

### 3.6. Case 4: NSCLC Stage III Bulky N2 Non-Oncogene-Addicted

A male patient, 78 years old, was diagnosed with stage IIIB (cT3N2) squamous cell lung carcinoma, presenting with a bulky right paratracheal region lymph node. The patient was in fair clinical condition with comorbid chronic obstructive pulmonary disease (COPD) and chronic heart failure. All three decision-making points in this case focused on the therapeutic management. The discussions were generally brief and extremely collaborative across all groups. A complete homogeneity was reached in the first decision point regarding the therapeutic choice, which was predominantly based on scientific evidence. The final decisions were shared by all the specialists involved in most groups; only in two groups was the decision primarily guided by a single individual.

The second decision point was discussed even more rapidly and was likewise characterized by strong agreement and collaboration in the therapeutic choices.

Overall, this case was marked by the highest level of consistency and collaboration among all those discussed, both in terms of content of the decision and the participatory nature of the discussion. The total time dedicated to each decision point was notably shorter compared to the other clinical scenarios. In particular, the third decision point was primarily led by the medical oncologist and pulmonologist. A graphical summary is provided in Figure 5; we resume the clinical case discussion.

## 4. Discussion

The management of lung cancer has undergone significant transformation in recent years, largely driven by the emergence of integrated therapy approaches [12,30]. Advances in molecular profiling, targeted therapies, immunotherapy, and precision radiotherapy [31] have redefined treatment paradigms, particularly for NSCLC [30,32]. These developments have significantly increased the complexity of treatment decision making, particularly in stage III NSCLC, reinforcing the need for a multidisciplinary approach to ensure that therapeutic strategies are both evidence-based and tailored to individual patients’ clinical features and molecular profiles [22]. In this context, the “Integrate 6.0” conference provides insight into the practical implementation of multidisciplinary discussion (MDD), in contemporary clinical practice. Findings from this education experience are consistent with previous reports highlighting the central role of multidisciplinary tumor boards in optimizing treatment strategies and improving clinical outcomes [10,33,34]. MDD plays a pivotal role, particularly in the management of complex NSCLC cases. The structured involvement of thoracic surgeons, medical and radiation oncologists in the analysis of clinical scenarios provided a valuable platform for integrating different perspectives. By facilitating collective decision making, MDD enables the treatment plan to be adapted to each patient’s unique clinical and molecular contexts, potentially improving outcomes [20,30,34], as previously reported in similar scenarios [34].

Our results showed variability in decision making across groups, particularly in the initial decision points of more complex clinical scenarios. As observed in the case-based discussions, consensus was less frequently achieved in early decision steps, whereas a higher degree of agreement was observed in subsequent decisions. This finding reflects the complexity of initial treatment selection and the influence of different specialist perspectives.

The clinical cases presented during the meeting highlighted the complexity of managing locally advanced NSCLC stages, particularly in those cases involving driver mutations (e.g., EGFR-positive, stage IIIB) or challenging nodal disease (e.g., bulky N2 disease). These findings align with the existing literature, including recent consensus efforts such as the EORTC survey on resectability [13], which highlighted the heterogeneity of stage III disease and the variability in clinical decision making across institutions. For instance, the integration of molecular profiling into treatment decisions highlighted the importance of personalized approaches, particularly when targeted therapies and immunotherapy options coexist [16,20,30,35].

The interactive, case-based format adopted during the meeting further emphasized the value of structured multidisciplinary dialogue. By simulating real-world tumor board dynamics, this approach facilitated the integration of different clinical perspectives and enhanced participants’ understanding of the rationale underlying complex treatment decisions. While similar educational strategies have been described, this report provides a detailed description of a structured, case-based multidisciplinary format specifically applied to stage III NSCLC management. The discussion of individual clinical scenarios highlighted several key challenges in current practice. The increasing use of neoadjuvant chemoimmunotherapy is reshaping surgical strategies, while the management of patients with oncogene-driven tumors continues to require careful integration of molecular and clinical data [36,37]. Similarly, the use of concurrent chemoradiotherapy followed by immunotherapy in unresectable disease reflects evolving standards of care, but also introduces new challenges, including the management of immune-related toxicities. These observations are consistent with emerging evidence from recent clinical trials and real-world studies. Despite these strengths, the present study has several limitations. First, the sample size was relatively small and participants were selected through a non-random process, which may introduce selection bias. Second, this study reflects an educational experience and does not include patient-level outcomes, limiting the ability to draw conclusions on clinical effectiveness. Finally, the findings may be influenced by geographic and organizational factors, potentially limiting generalizability. Overall, this study supports the growing evidence that multidisciplinary collaboration is essential in the management of stage III NSCLC and highlights the educational value of structured, case-based discussion formats. Future studies are warranted to evaluate the impact of such approaches on clinical decision making and patient outcomes, in which participants were divided into several multidisciplinary groups to independently discuss clinical cases, effectively demonstrating the practical benefits of collaborative teamwork.

## 5. Conclusions

In conclusion, the “Integrate 6.0” conference highlights the importance of multidisciplinary discussion in the management of stage III NSCLC, supporting its role in facilitating complex clinical decision making in an evolving therapeutic landscape. This educational experience reinforces the value of structured, case-based interactions in promoting collaboration among different specialties and improving the integration of evidence-based and personalized treatment strategies. However, the findings should be interpreted in light of several limitations, including the relatively small sample size, the non-random selection of participants, and the absence of patient-level outcome data. Future initiatives may benefit from longitudinal assessment of how MDD influences patient outcomes and from expanding the multidisciplinary panel to include additional specialties, such as molecular pathologists and geriatricians, thereby enriching the decision-making process even further. By combining interactive case-based discussions with focused scientific updates, the “Integrate 6.0” conference provides a structured educational model that may support multidisciplinary clinical reasoning in the management of locally advanced stage III NSCLC.

## Data Availability

The data supporting the findings of this study are available from the corresponding author upon reasonable request.

## Supplementary Materials

The following supporting information can be downloaded at: https://www.mdpi.com/article/10.3390/jcm15103752/s1, Supplement S1: Supplement Clinical cases, Figure S1: Supplement S2 Geographical distribution and specialization of the survey participants, Figure S2: Supplement S3 Geographical distribution and affiliation of the survey participants, and Figure S3: Supplement S4 Participants’ professional experience and the proportion of their time dedicated to lung tumor treatment.
