# Supplementary material for: Multidisciplinary Decision-Making and Integrated Strategies in Stage III Non-Small Cell Lung Cancer: Exploring Clinical Reasoning in Therapeutic Choices
Source: J Clin Med. 2026 May 13;15(10):3752. doi: 10.3390/jcm15103752 (PMC13206792; doi:10.3390/jcm15103752)
Supplement: Supplementary file 1 [file jcm-15-03752-s001.zip › jcm-4221240-supplementary/Supplement File S1.pdf]

SUPPLEMENT CLINICAL CASES

Clinical case and decision-making points.

Case 1: NSCLC CT4N0M0 eligible to pneumonectomy

A 69-year-old male patient, former smoker with a 30-year history, presented with a medical history of chronic ischemic heart disease, arterial hypertension, dyslipidaemia, and hiatal hernia. His performance status was ECOG 0. The patient was admitted to the emergency department due to the onset of a persistent dry cough, associated with occasional nausea and mild weight loss (approximately 5 kg). A chest X-ray followed by contrast-enhanced chest CT revealed a large mass measuring 10 cm × 7.2 cm in the left lower lobe, suggestive of a pulmonary neoplasm. FDG PET-CT confirmed the pulmonary lesion, with no evidence of suspicious hilar or mediastinal lymphadenopathy. Endobronchial ultrasound-guided transbronchial needle aspiration (EBUS-TBNA) of mediastinal lymph nodes (stations 4R, 10R, and 7) confirmed the absence of nodal involvement. A biopsy of the pulmonary lesion was performed, and histological examination revealed a poorly differentiated adenocarcinoma with PD-L1 expression of 5%. Next-generation sequencing (NGS) identified a KRAS G12C mutation. Contrast-enhanced brain CT ruled out secondary lesions, and pulmonary function tests were within normal limits.

1st decision-making point: What therapeutic approach would you propose?

A) Pneumonectomy

B) Concurrent chemoradiotherapy
C) Neoadjuvant chemotherapy followed by surgery
D) Neoadjuvant chemoimmunotherapy followed by surgery

Following multidisciplinary discussion, the patient was proposed for neoadjuvant chemoimmunotherapy, pending cardiological evaluation and a pre-surgical staging CT scan. Treatment was initiated with carboplatin, pemetrexed, and nivolumab. The patient developed grade 3-4 cutaneous toxicity, which was managed with corticosteroids and antihistamines. Restaging CT showed a mild response to systemic therapy, with tumour dimensions reduced to 7.9 × 6.0 mm. No secondary lesions or pathological lymph nodes were detected.

2nd decision-making point: What local treatment should be proposed?

A) Pneumonectomy
B) Lobectomy, if feasible
C) Definitive radical-dose radiotherapy
D) D) Additional three cycles of chemoimmunotherapy

The patient underwent left upper lobectomy and lymphadenectomy. Histological examination revealed a large-cell neuroendocrine carcinoma (pT4pN0) with negative surgical margins.

3rd decision-making point: What should be the next step in management?

A) Adjuvant chemotherapy
B) Adjuvant chemoradiotherapy
C) Adjuvant radiotherapy
D) Adjuvant immunotherapy

Postoperative staging CT showed no evidence of residual disease. After multidisciplinary discussion, the patient was scheduled for adjuvant chemotherapy with carboplatin and etoposide.

Case 2: NSCLC cN2 multistation with PD-L1 >50%

A 65 year old woman, never smoker, presented with cough and haemoptysis and subsequently underwent a chest x-ray ad CT scan with contrast, leading to a diagnosis of lung tumour in the right lower lobe, measuring 5.3 cm, with enlarged lymph nodes at station 7 and 8. Brain and abdominal CT scans were negative for metastases, while 18-FDG PET showed pathological uptake at the tumour site and in the hilar, station 7 and 8 lymph nodes.

Bronchoscopy with integrated EBUS/EUS for mediastinal staging confirmed the presence of primitive lung adenocarcinoma with pathological involvement of stations 10, 7 and 8, resulting in a clinical stage of cT3N2M0.

1st decision-making point: What additional analysis should you perform on the biopsy?

A) None
B) PD-L1 alone
C) EGFR, ALK, BRAF, K-RAS, ROS-1, PD-L1
D) NGS alone

Additional evaluation included EGFR, ALK, BRAF, K-RAS, ROS-1 and PD-L1 analysis.

The patient was subsequently evaluated by the multidisciplinary lung unit, and the surgeons deemed the tumour potentially resectable with right lower lobectomy and lymphadenectomy.

2nd decision-making point: What treatment should be proposed?

A) Upfront surgery

B) Induction therapy followed by surgery

C) Definitive chemoradiotherapy

D) Immunotherapy

The multidisciplinary board decided/opted for induction therapy followed by surgery. After four cycles of platinum-based chemotherapy, the patient was re-evaluated. CT scan showed tumour and lymph node shrinkage, and 18-FDG PET showed/indicated/demonstrated a complete nodal response and a partial tumour response.

A uniportal VATS right lower lobe lobectomy was performed, integrated with mediastinal radical lymphadenectomy (stations 11,10,2,4,7,8,9). The final pathological report confirmed complete resection of a 3,7 cm G3 solid adenocarcinoma with 55% viable tumour cells (according to QU classification), pleural infiltration (PL2) and metastases in two station 11 lymph nodes. The pathological stage ypT2N1M0.

3rd decision-making point: What further program should be proposed?

A. Follow-up

B. Adjuvant chemotherapy

C. Immunotherapy

D. Adjuvant radiotherapy

Case 3: NSCLC stage III multiple N2 with driver mutation

A 68 -year-old male patient with a family history of colon-rectal cancer. He is a never smoker and former railway worker with occupational exposure to asbestos. His medical history includes benign prostatic hyperplasia and occupational hearing loss. His performance status is ECOG 0. Following persistent cough and haemoptysis unresponsive to antibiotic therapy, a chest X-ray was performed, followed by a chest CT scan, which revealed a suspected right-side mass. The chest CT scan revealed a gross measuring 60x47x42 mm in the dorsal segment of the right upper lobe (RUL) along with multiple partly confluent lymphadenopathies in the right hilum (Station 10R) and pre- and subcarinal regions (Stations 7 and 4R) with a maximum short axis of 18 mm. An 18-FDG PET scan was subsequently conducted, confirming the presence of neoplastic tissue with high glucose uptake in the right upper lobe and 4R, 10R and 7 lymph nodes.

The patient underwent Endo Bronchial Ultrasound (EBUS) with biopsy at station 4R, which provided histological evidence of adenocarcinoma with EGFR mutation (exon 21 pL858M) detected via NGS and negative PD-L1 expression. The final

staging was T4N2M0 Stage IIIB according to the 8th AJCC classification.

1st decision-making point: Before proceeding the therapeutic decision, which diagnostic process would be useful as a complement?

A) Brain MRI
B) Bone scintigraphy
C) Pulmonary function test
D) Comprehensive cardiac evaluation

2nd decision-making point: While waiting the results of the LAURA study, which therapeutic approach would be most appropriate?

A) Osimertinib
B) Surgery followed by chemotherapy and Osimertinib
C) Chemoradiotherapy followed by clinical-radiological follow-up
D) Chemoradiotherapy and re-biopsy for re-evaluation of PD-L1

Following a multidisciplinary discussion, the patient was deemed unsuitable for surgery. Instead, the patient underwent thoracic radiotherapy with a total dose of 60 Gy in 30 fractions in combination with chemotherapy consisting of Cisplatin (75 mg/m2) and Vinorelbine (25 mg/m2) for three cycles. Subsequently, the patient continued treatment with pembrolizumab as part of the MPLALC clinical study for 12 cycles. One year after diagnosis, the patient developed a single intracranial progression, leading to the discontinuation of the study.

3rd decision-making point: In case of single brain oligoprogression, during maintenance immunotherapy (clinical study) in a patient with EGFR-positive lung adenocarcinoma, the most appropriate approach would be:

A) Stereotactic brain radiotherapy and continuation of ongoing immunotherapy
B) Stereotactic brain radiotherapy followed by initiation of Osimertinib
C) Initiation of Osimertinib, with Stereotactic brain radiotherapy in the event of clinical and radiological progression
D) Stereotactic Brain radiotherapy alone

The patient underwent stereotactic radiotherapy for a single brain lesion and was simultaneously initiated on Osimertinib.

Case 4: NSCLC stage III bulky N2 non-oncogene-addicted

A 78-year-old, male, former smoker (40 packs/yr) underwent a CT scan of the thorax following haemoptysis, which revealed a

right hilar mass of 56 x 51 mm. The patient was in good clinical conditions (ECOG PS 1). A bronchoscopy was performed, revealing a diagnosis of squamous cell carcinoma of the lung (p63 positive, TTF-1 negative) with high PD-L1 tumour expression (Clone SP263, TPS ≥50%). Disease staging via CT/PET determined cT3N2M0 – Stage IIIB (TNM VIII ed.) due to the presence of bulky lymph node metastases in the right hilar and subcarinal regions. The mass caused *"ab estrinseco"* compression of the main right bronchus, leading to atelectasis of the right upper lobe.

A self-expanding prosthesis was implanted into the main right bronchial system. The case was subsequently discussed by a multidisciplinary tumour board.

1st decision-making point: What treatment should be proposed?

A) Sequential chemo-radiotherapy followed by durvalumab consolidation in the absence of disease progression
B) Concurrent chemo-radiotherapy, followed by durvalumab consolidation in the absence of disease progression
C) Induction chemotherapy and then surgical resection
D) Cemiplimab monotherapy

After the multidisciplinary discussion, the patient was deemed unresectable and eligible for concurrent chemo-radiotherapy (cCRT). The patient underwent radiotherapy on the thoracic disease and lymph nodes with a total dose of 60 Gy in 30 fractions concomitant with platinum-based chemotherapy (paclitaxel 45 mg/m^2 plus carboplatin AUC2 weekly for 6 weeks). The treatment was well tolerated, except for esophagitis G2 at the end of the cCRT, which resulted in <10% weight being managed with nutritional supplementation and intravenous hydration. A CT scan performed 3 weeks from cCRT completion showed a partial response as per RECIST 1.1 criteria (right hilar lesion reduced from 56x51 mm vs. 31x35 mm).

2nd decision-making point: What further treatment should be possible?

A) Initiate durvalumab following complete resolution of radiation-induced esophagitis
B) Follow-up, omitting durvalumab consolidation treatment
C) C) Re-evaluate the feasibility of surgical resection
D) Administer consolidation chemotherapy

After a second MTB discussion and adequate recovery from cCRT, durvalumab 10 mg/kg every 2 weeks was started. The patient experienced rapid clinical improvement with progressive weight gain, absence of disease-related symptoms and no side

effects, with an ECOG PS 0. A CT scan after 4 courses of durvalumab revealed a slight increase of the right hilar mass (48 mm vs. 35 mm in maximum diameter) associated with honeycomb-like parenchymal consolidation in the right upper lobe and mild ipsilateral pleural effusion. The patient was completely asymptomatic with a normal pulse. A new MTB discussion was planned to differentiate immune-related pneumonitis or radiation-induced pneumonitis, or disease progression.

3rd decision-making point: How should this possible side effect be managed?

A) Initiate oral steroids and antibiotics, followed by a repeat CT scan in 4 weeks; restart durvalumab if resolution was achieved
B) Permanently discontinue durvalumab
C) Continue durvalumab without modification
D) Continue durvalumab with the addition of low-dose oral steroids

Based on radiographic and clinical findings, grade 1 immune-related pneumonitis was suspected. Durvalumab was temporally stopped, and a course of oral steroids and antibiotics was commenced, as an infectious cause could not be excludible. Oral steroids were gradually tapered. A follow up CT scan of thorax at two weeks showed a partial resolution of the parenchymal consolidation and pleural effusion, which had completely resolved after 4 weeks. Durvalumab was subsequently resumed and the patient completed 1-year consolidation immunotherapy without significant adverse events or recurrence of. The patient is disease free at more than 4 years after the initiation of cCRT.
