# Supplementary figures and images for: Multidisciplinary Decision-Making and Integrated Strategies in Stage III Non-Small Cell Lung Cancer: Exploring Clinical Reasoning in Therapeutic Choices
Source: J Clin Med. 2026 May 13;15(10):3752. doi: 10.3390/jcm15103752 (PMC13206792; doi:10.3390/jcm15103752)

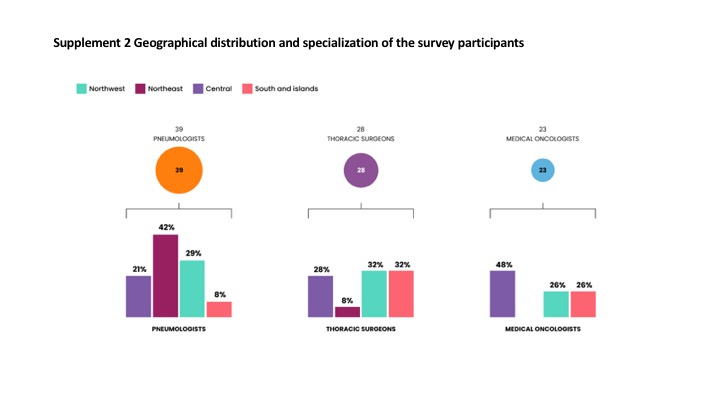

Supplement: Supplementary file 1 [file jcm-15-03752-s001.zip › jcm-4221240-supplementary/Figure S1.jpg]

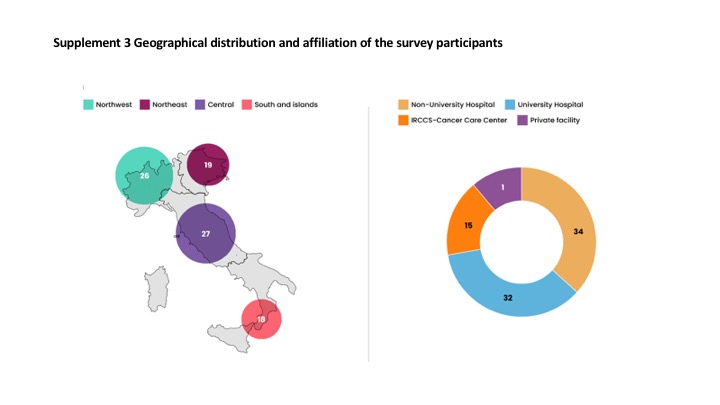

Supplement: Supplementary file 1 [file jcm-15-03752-s001.zip › jcm-4221240-supplementary/Figure S2.jpg]

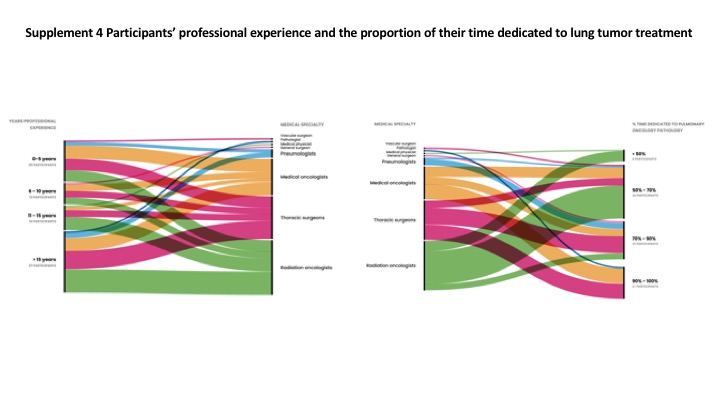

Supplement: Supplementary file 1 [file jcm-15-03752-s001.zip › jcm-4221240-supplementary/Figure S3.jpg]
